# Case Report: A new variant of the forehead flap for subtotal nose reconstruction in a single stage: the dragonfly flap

**DOI:** 10.3389/fsurg.2024.1420673

**Published:** 2024-08-09

**Authors:** Tito Brambullo, Arianna Franchi, Giuseppe Masciopinto, Alberto De Lazzari, Vincenzo Vindigni, Franco Bassetto

**Affiliations:** Plastic Surgery Unit, Department of Neurosciences, School of Medicine and Surgery, University of Padua, Padua, Italy

**Keywords:** forehead flap, nasal reconstruction, rhinoplasty, surgical technique, skin cancer

## Abstract

**Background:**

The forehead flap is probably the most used method for nose reconstruction after cancer resection. During the past century, this technique has been continuously refined to achieve better functional and aesthetic outcomes. Different variations have been described, with the original technique being modified based on tissue loss, the layer to be replaced, and the management of the donor area.

**Methods:**

We propose a new and innovative version of the forehead flap in which both the forehead skin and the frontal muscle are harvested simultaneously using the same vascular pedicle. Partially separating the two layers allows muscle tissue to replace the inner layer and cover the nasal septum framework, while the skin will replace the outer layer. The nostrils are reconstructed simultaneously using bilateral hinge-over lining skin flaps harvested from the nasal folds.

**Results:**

Step by step, a schematic illustration of the technique is given, followed by a complete report on a successful total nose reconstruction case.

**Conclusions:**

Despite the increasing number of techniques which have been introduced to achieve full reconstruction of the nose, including microsurgical tissue transfer, the simultaneous replacement of both the inner and outer layers continues to be an issue for the plastic surgeon. In this article, we suggest a solution for total nose reconstruction in a single-stage procedure.

## Introduction

Since the 6th century when Sushruta first introduced the method of nasal reconstruction with a forehead flap (1, 2), the original technique has remained largely unchanged. Its remarkable adaptability to various losses of tissue, combined with its ability to replicate the native skin texture and color of the nasal pyramid, has made it an enduring and effective solution.

Over several centuries, the fundamental principles of reconstruction have remained constant: it is necessary to replace all three layers of the nose to avoid distortions and impairments to airflow while achieving a satisfactory cosmetic outcome. These three layers are comprised of an internal lining, a bone/cartilage structure, and an outer skin envelope (3).

Regarding the primary objective of repairing external skin loss after skin cancer ablation, the forehead flap has proven to be an exceptionally effective and dependable solution (4). On the other hand, addressing the need to cover underlying structures, such as the cartilaginous septum, and providing suitable support for cartilage grafts typically requires the transposition of other mucosal or cutaneous flaps (5–7).

Complex reconstruction of this nature is usually accomplished through a multistage procedure, requiring the patient to undergo multiple operations to achieve the final result (3). To minimize the need for additional procedures, we explored the feasibility of reconstructing the outer and inner soft tissue layers of the nose using only the forehead flap. The extensive blood supply branches present throughout the skin and muscle tissues of the frontal region make aggressive dissection a viable and safe option (8).

By partially separating the muscular component from the skin paddle, the former can be used to encase and nourish the cartilaginous framework or grafts, while the latter is responsible for the outer covering.

In this article, we introduce our preliminary results with such innovative technique.

## The “dragonfly” flap technique

Upon the completion of nasal cancer excision, various anatomical structures may be absent, such as the nasal skin from the nasion to the labial philtrum, the alar and triangular cartilages, the cartilaginous septum, the proximal bone septum, and a variable portion of the nasal fold skin.

**Figure 1:**
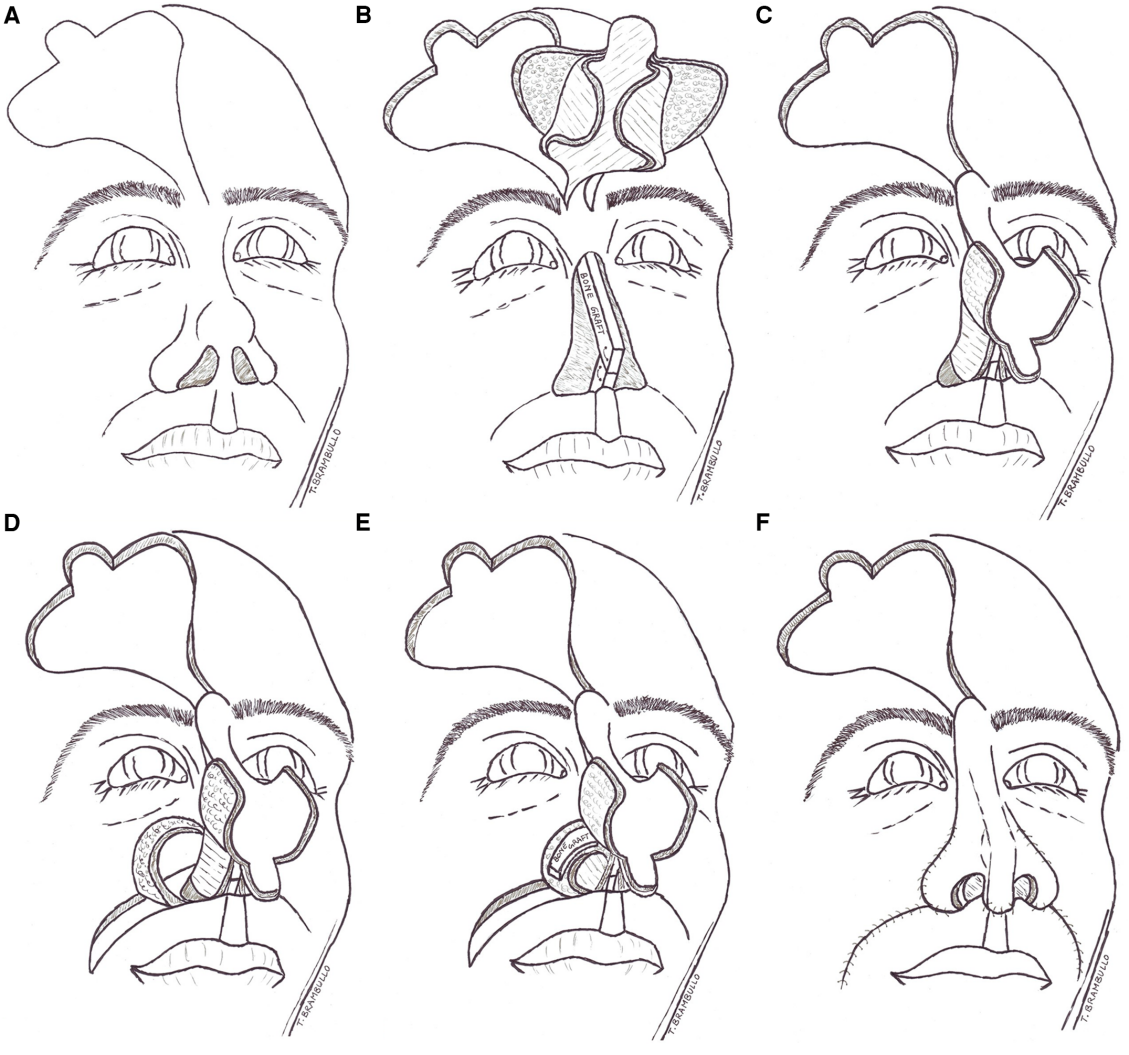
Schematic illustration of the dragonfly technique. The conventional forehead flap design (**A**) Composite flap harvested and split and nasal resection with cantilevered septum reconstruction complete (**B**) Transposition of the flap on defect and complete covering of septum framework with the flap muscle wings (**C**) Sculping of the hinge-over skin flaps from the labial folds (**D**) Grafting of neo-alars with bone or cartilage (**E**) Final external covering of alars with the flap lateral cutaneous wings (**F**).

The preoperative part of the procedure begins by measuring the size of the skin defect, and its representation on the forehead and flap axes is determined based on the width and height of the hairless skin (Figure 1A).

The forehead flap is harvested at full thickness, encompassing the underlying frontal muscle, while leaving only the periosteum above the calvaria. The dissection proceeds from the capillitium margin towards the origin of the supratrochlear vessels at the medial third of the eyebrow. Approximately 1 cm above the eyebrow, dissection proceeds subperiosteally. The forehead flap is then harvested in a conventional manner.

At this point, both lateral portions of the frontal muscle are gently dissected from the overlying skin paddle while carefully keeping the two layers in contact for a width of 1 cm along the flap axis. Consequently, the flap is partially divided into four sections, resembling a four-winged dragonfly (Figure 1B).

The so-shaped flap is then advanced to cover the prelaminated cartilage or bone framework that replaces the nasal septum. The two muscle wings of the flap are bended to cover both sides of the framework instead of the removed native mucosa. To maintain the proper positioning of the muscle flaps and eliminate any dead space between them, some sutures are used to connect them side to side (Figure 1C).

Two hinge-over flaps are obtained from each nasal fold and transferred medially to the reconstructed septum, particularly in cases of complete alar loss (Figure 1D). To prevent alar collapse during inspiration, a piece of cartilage or bone is typically grafted onto the subcutaneous surface, while the skin paddle forms the inner nostril vault (Figure 1E).

After the completion of the procedure, the forehead flap's skin wings are securely sutured over the alar grafts, and the tip of the flap is anchored to the labial philtrum, effectively reconstructing the columella (Figure 1F). The entire single-stage nose reconstruction has been accomplished. It is essential to carefully position the nostril splints to prevent excessive internal pressure.

The removal of sutures will be determined by the healing of the wound, and the sectioning of the flap pedicle will be scheduled 3 to 6 weeks following the surgery.

## Case description

A 54-year-old female patient presented at our outpatient clinic with recurrent basal cell carcinoma of the nasal skin.

She had previously undergone several partial excisions of the cancer with no free margins. The patient's clinical history was unremarkable for any other disease.

**Figure 2:**
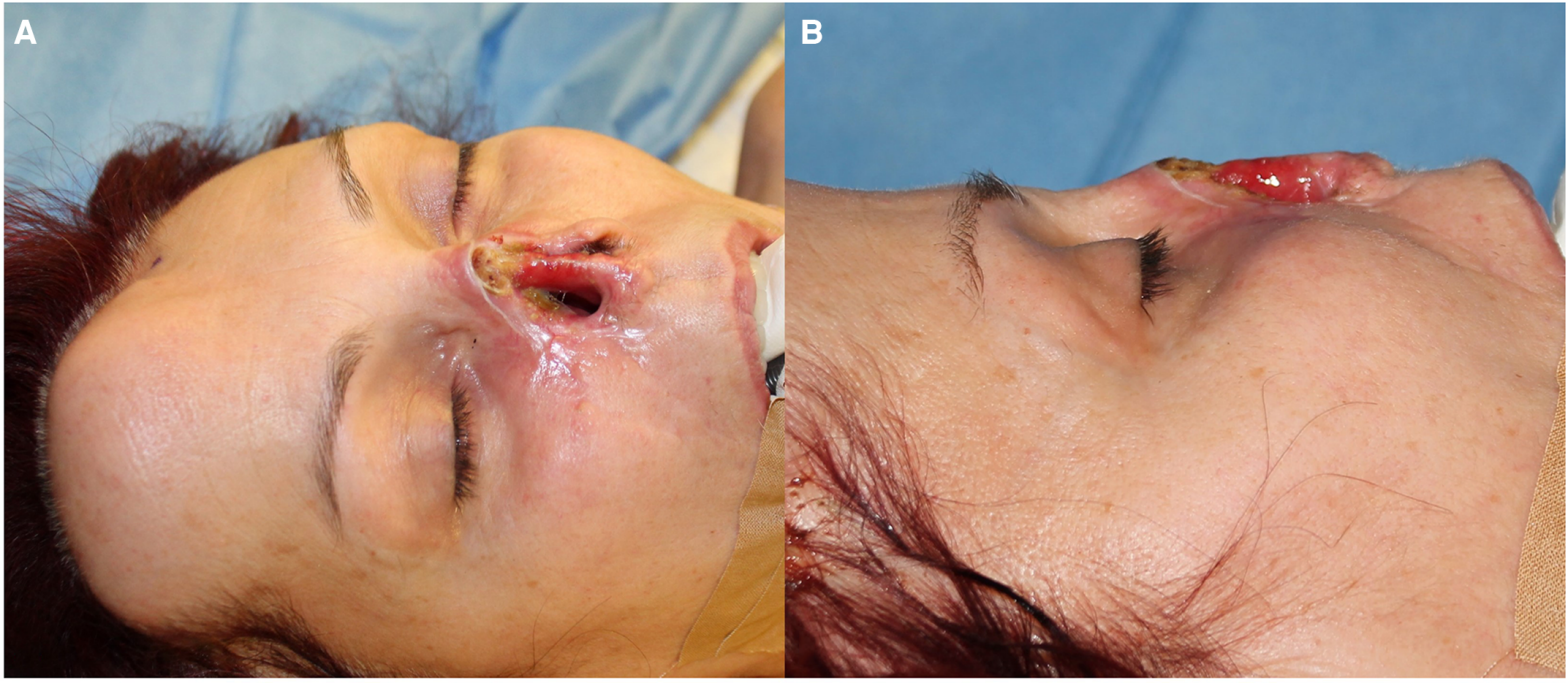
The local conditions following the eradication of basal cell cancer. The entire nasal pyramid and a part of the septum are absent, frontal view (**A**) and lateral view (**B**).

We discussed the pros and cons of a procedure for tumor eradication without immediate reconstruction to wait for the pathologist's response regarding the margins. Full-thickness resection of the external skin envelope of the dorsum and right alar was performed, but the left and inferior skin margins and nasal septum were still involved in the cancer. In the second surgery, all residual nasal subunits were removed, including the columella, the entire cartilaginous septum, and the distal part of the bone septum (Figure 2). In the same procedure, a tissue expander was positioned under the scalp and hairless frontal area to increase the flap surface and allow direct donor site closure. Unfortunately, the tissue expander was subsequently exposed through the skin incision and had to be removed.

Finally, the resection margins were disease-free, so a reconstructive procedure was planned.

Given the limited amount of nasal mucosa, microsurgical reconstruction of the inner layer was proposed to ensure adequate coverage of the septum to be reconstructed. However, the patient declined this treatment option and requested a single-stage procedure.

This complex situation necessitated a reevaluation of regional surgical options and the anatomical pathway of the supratrochlear vessels. The extensive distribution of source vessels within the substance of both the skin and muscle led to the planning of a composite forehead flap that allows for the concurrent replacement of the inner and outer nasal layers.

Preoperatively, measurements were taken to determine the amount of skin required to cover the nasal fossae and provide an adequate height for the dorsum and tip of the nose.

In the third surgical procedure, a cantilever bone graft was created by combining two sections of rib harvested from the sixth and seventh left ribs. This graft was then modeled and fixed in a framework that served as a new septum.

**Figure 3:**
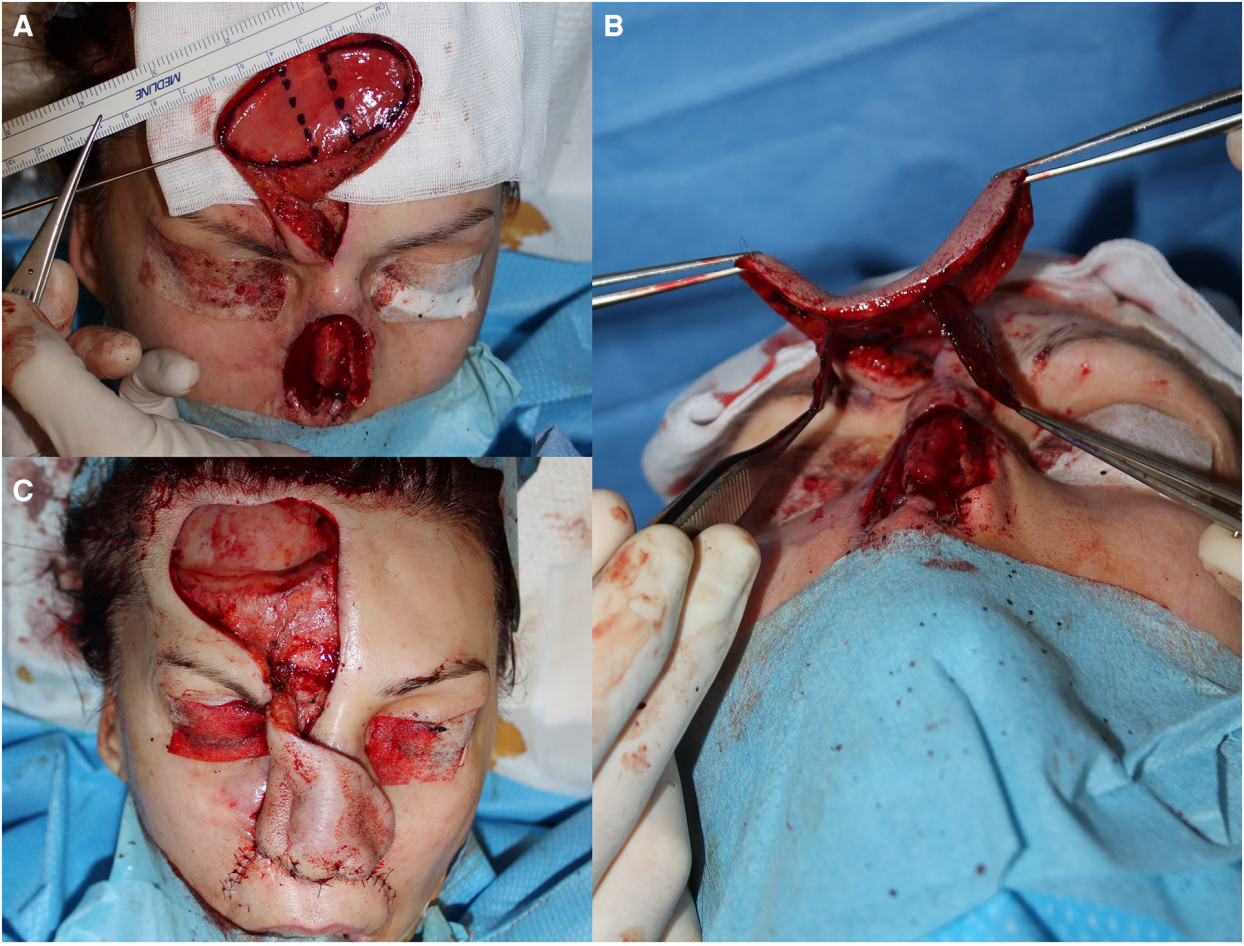
The harvest of the composite forehead flap (**A**) splitting the flap's edges into four wings resembling the dragonfly (**B**) after that, the flap is rotated to cover the septal cantilever graft (**C**).

During the procedure, a conventional design for a forehead flap was drawn for total nose replacement. A full-thickness composite flap, which included the muscular tissue of the underlying frontal muscle, was then harvested. The dissection of the flap into four wings proceeded as previously described, and the flap was transposed to cover the septal framework (Figure 3). Additionally, two cutaneous hinge-over flaps were harvested from each nasolabial fold and rotated to the neoseptum to recreate the nostrils.

Two cartilage grafts were positioned and secured to the hinge-over flaps to prevent collapse during inspiration. Since the patient preferred not to wait for the second intention of healing, the donor area of the forehead was grafted with skin. Finally, the final sutures were applied, and nasal stents were positioned.

The duration of the surgical procedure was 3.5 h, and the patient experienced an uncomplicated postoperative period. After three days, the patient was discharged and the splints were removed after five days.

The external sutures were removed after twelve days, and the pedicle sectioning was planned and executed after four weeks.

**Figure 4:**
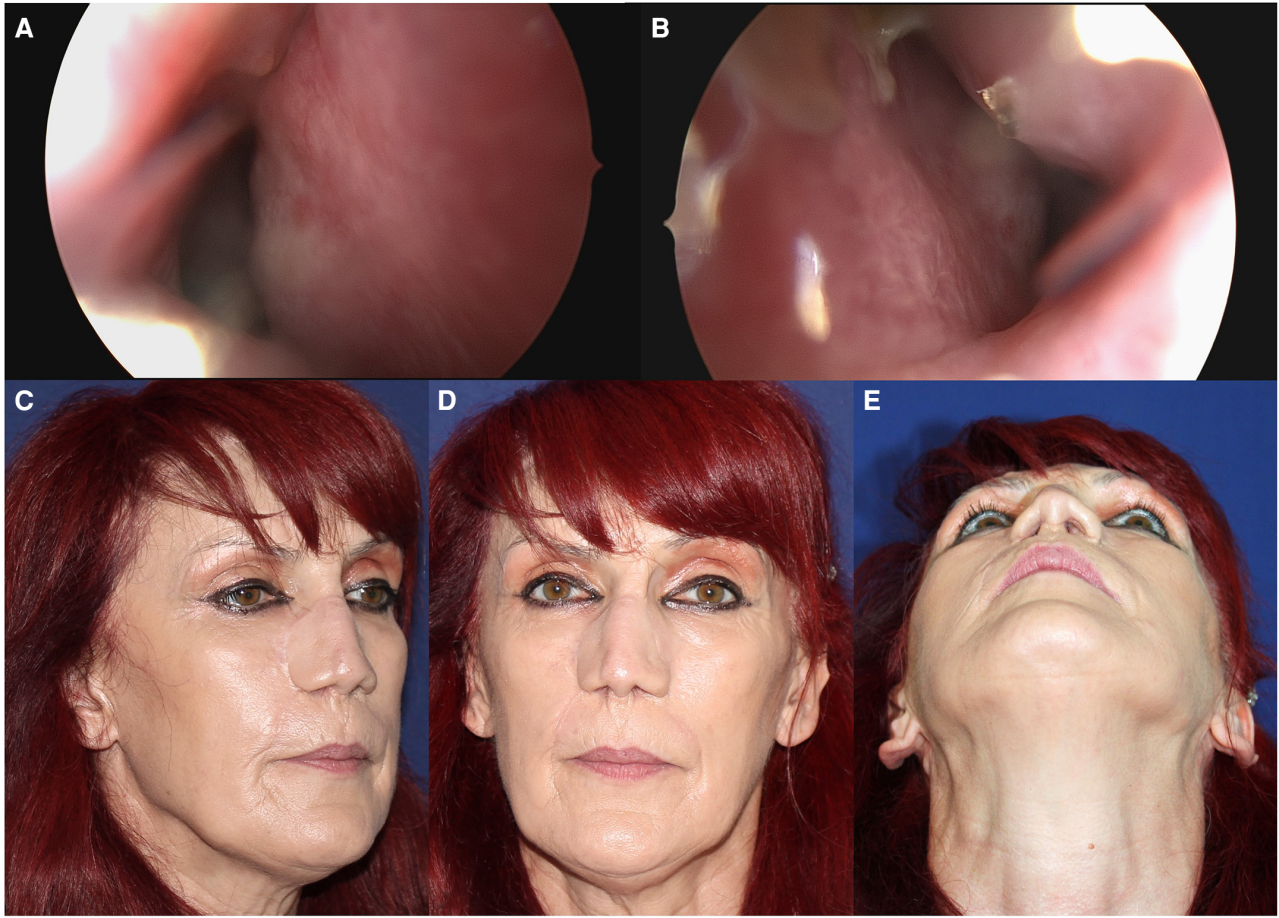
An endoscopic optical device is used for a 40-day postoperative endonasal check. There was no fistula or residual bare muscular surface present on either side of the neo-septum. The right side (**A**) and the left side (**B**) Aesthetic outcome after 6 months, lateral (**C**) and frontal view (**D**) and the view from below (**E**).

During the forty-day postoperative follow-up, the patient's septum, which had been reconstructed with three laminated layers, was examined using an endoscopic optical device (Figures 4A,B). A new, mature mucosal layer with a normal appearance covered the entire surface of the forehead flap's muscular wings. No pathological holes or interruptions in the septum were observed.

Three months after the initial procedure, the patient underwent another surgery to refine the dorsum and increase the nasal tip projection, which was achieved through cartilage grafting from the auricular concha. The patient was advised to wear nostril splints during this period to prevent deformation caused by scarring.

Following this surgery, the patient reported being satisfied with both the functional and aesthetic outcomes (Figures 4C–E).

There were no signs of vascular impairment after any of the procedures, and the patient did not report any airflow disturbance or alar collapse during outpatient visits or otherwise.

## Discussion

Reconstruction of the nose is widely regarded as one of the most complex plastic surgery procedures due to the intricate process of replicating the various layers of tissue in their natural proportions. This delicate task requires a high degree of skill and precision, as any miscalculation or error can result in the formation of irregularities, airflow impairment, and aesthetic concerns (9, 10).

Although there is still no definitive solution to the problem, several variations of the original technique of forehead flap have been found to be effective in achieving better outcomes (11, 12).

However, the majority of the literature is focused on nasal partial reconstruction, which involves reconstructing only a full-thickness missing subunit or proposing solutions for the entire external skin layer without considering the issues of the internal lining and cartilage framework (13–15). In the most challenging situations, where the skin and its underlying layers necessitate subtotal replacement, reconstructive measures typically entail harvest and transposing of multiple local flaps in conjunction with septal or alar grafting (16, 17). An alternative approach involves microsurgical tissue transplantation of any required size, which allows for prelamination of composite flaps with cartilage or bone grafting before transferring them to the defect. This approach has been shown to be effective in restoring the natural contour of the face, and can be used to treat a variety of defects, including those caused by trauma, surgery, or congenital abnormalities (18). Even if this can be considered the ultimate solution to address otherwise unsolvable problems, some authors have posed evidence of aesthetic limitations of such reconstruction, urging the use of a forehead flap to cover a microsurgically rebuilt nose (19).

Another issue is the number of stages required to complete reconstruction, from the patient's perspective, fewer procedures with less complexity are generally preferred whenever possible.

In this scenario, the dragonfly forehead flap was thought to be a reliable solution for the loss of the entire external nasal vault, along with the septum and alar framework, with the advantage of completing the entire reconstruction in one stage.

However, it is still necessary to detach the pedicle after a few weeks, as in the traditional approach. Some authors have suggested a modification that involves thoroughly dissecting the subcutaneous tissue of the pedicle to bury it, thereby avoiding the external route and eliminating the need for sectioning (20, 21).

Additionally, Cordova et al. proposed a reliable propeller flap based on a supratrochlear perforator, which eliminates the need for a second stage (22).

Compared to other techniques, this solution allows for the simultaneous treatment of wider soft tissue defects and the absence of a dorsal septum and columella.

A potential alternative to this method would be a half-shaped dragonfly flap, which involves harvesting the paramedian forehead flap using only one muscular wing. This approach may prove beneficial in cases where the cancer has only invaded one side of the nasal vault, necessitating asymmetric resection, as in the case of cancer arising from the inner mucosal layer; however, we have not yet experienced this.

Additionally, it permits the entire procedure to be carried out using a single donor site, the forehead, with the exception of the labial fold, from which the first author's preferred flap for nostril reconstruction is harvested.

The use of two additional hinge-over flaps have proven to be of great benefit in improving the natural-like appearance of the neo-nostrils whenever the defect involves completely one or both alars; therefore, we have included this flap together with the use of alar grafting in the description of the technique as an integral part of the procedure.

The limitations associated with nasal reconstruction utilizing a forehead flap may not be entirely resolved through this technique. Potential outcomes such as donor site healing complications, suboptimal cosmetic results, and eventual deformities may still occur.

The process of regenerating the nasal mucosa over the muscular surfaces of the wings, which cover both sides of the reconstructed septum, presents another disadvantage. During this period, the septal framework is prone to exposure and collapse due to partial or complete necrosis of the muscular wings. To investigate this issue, we conducted an endoscopic examination of both nasal fossa 40 days after the procedure. The results of our examination revealed that the muscle wings had completely regenerated without any evidence of fistulas, confirming the efficacy of the surgical procedure.

A similar variant of this approach has been described by Harrison et al. (23), in that report a chimeric forehead flap was harvested, composed of a pericranium layer flap and the conventional skin paddle, using the supratrochlear vessels as a common blood source. The first served as a nourishing bed for the rib grafts, while the latter was used for external covering.

The primary distinction between this method and ours pertains to the components of the chimeric flap. The risk of vascular impairment is substantial when harvesting a pure periosteum flap, as its blood supply is difficult to accurately determine and safely preserve. Secondly, the placement of the pericranium flap under the graft may restrict its range of motion and consequently impede complete coverage of the septum. Lastly, if the size of the forehead flap surpasses the possibility of direct closure, the calvaria would be uncovered at the donor site, which would significantly impact its potential application in the event of total nose loss.

Finally, it should be clearly stated that aesthetic enhancements of the nose profile can still be necessary after reconstruction with the dragonfly flap, since this approach does not offer any additional benefit with respect to the traditional procedures under this view.

The process of flap revision involves reopening a section of the suture line, removing excess fat from the flap, and reshaping the supra-alar convexity following the steps described in the literature (4).

## Conclusions

To the best of our knowledge, this is the first description of such a variant of forehead flap, and as with any new technique, further experience is needed to properly evaluate the pros and cons before drawing definitive conclusions.

The dragonfly flap is a promising new solution for restoring complete loss of the nose, reducing the overall impact of a complex procedure on the patient, and achieving favorable outcomes in terms of respiratory function and aesthetic appearance.

## Data Availability

The original contributions presented in the study are included in the article/Supplementary Material, further inquiries can be directed to the corresponding author.
